# Development of coronary artery stenosis in a patient with metastatic renal cell carcinoma treated with sorafenib

**DOI:** 10.1186/1471-2407-12-231

**Published:** 2012-06-11

**Authors:** Maria Abbondanza Pantaleo, Anna Mandrioli, Maristella Saponara, Margherita Nannini, Giovanna Erente, Cristian Lolli, Guido Biasco

**Affiliations:** 1 Department of Hematology and Oncological Sciences “L&A Seràgnoli”, S. Orsola-Malpighi Hospital, University of Bologna, Bologna, Italy; 2 “Giorgio Prodi” Cancer Research Center, University of Bologna, Bologna, Italy; 3 Laboratorio di Emodinamica, Struttura Complessa di Cardiologia (Direttore Dr Angelo Ramondo), Ospedale S. Bassiano, Bassano del Grappa, Verona, Italy; 4 University of Bologna, Department of Hematology and Oncological Sciences "L.A. Seragnoli", S. Orsola-Malpighi Hospital, Via Massarenti 9, 40138, Bologna, Italy

**Keywords:** Sorafenib, renal cell carcinoma, coronary syndrome, cardiotoxicity, cardiac ischemia/infarction

## Abstract

**Background:**

Tyrosine kinase inhibitors (TKIs) are currently approved for the treatment of metastatic renal cell carcinoma (mRCC). The cardiotoxic effects of sorafenib and sunitinib may cause hypertension, left ventricular ejection fraction (LVEF) dysfunction and/or congestive heart failure (CHF), and arterial thrombo-embolic events (ATE). Only three cases of coronary artery disease related to sorafenib therapy have been described in the literature, and all were due to arterial vasospasm without evidence of coronary artery stenosis on angiography. Cardiotoxicity is commonly associated with the presence of cardiovascular risk factors, such as a history of hypertension or coronary artery disease.

**Case presentation:**

We describe a patient who experienced an unusual cardiac event after 2 years of sorafenib treatment. A 58-year-old man with mRCC developed acute coronary syndrome (ischemia/infarction) associated with critical sub-occlusion of the common trunk of the left coronary artery and some of its branches, which was documented on coronary angiography. The patient underwent triple coronary artery bypass surgery, and sorafenib treatment was discontinued. He did not have any cardiovascular risk factors, and his cardiac function and morphology were normal prior to sorafenib treatment.

**Conclusions:**

Further investigation of a larger patient population is needed to better understand cardiac damage due to TKI treatment. Understanding the usefulness of careful cardiovascular monitoring might be important for the prevention of fatal cardiovascular events, and to avoid discontinuation of therapy for the underlying cancer.

## Background

Tyrosine kinase inhibitors (TKIs) are currently approved for the treatment of metastatic renal cell carcinoma (mRCC) [1,2]. Common toxicities due to sorafenib and sunitinib treatment include reversible skin rashes, hand-foot skin reaction, diarrhea, hypertension, hemorrhage, and laboratory findings such as leucopenia, hypophosphatemia, elevated pancreatic enzymes levels, and proteinuria [3,4]. Other clinical events related to cardiotoxicity include left ventricular ejection fraction (LVEF) dysfunction and/or congestive heart failure (CHF) and arterial thrombo-embolic events (ATE) [3-8]. Several authors have concluded that LVEF dysfunction and/or CHF is more frequent in patients with a history of hypertension or coronary artery disease. Only three cases of coronary artery disease related to sorafenib treatment have been described in the literature. All three cases were associated with the presence of cardiovascular risk factors and were due to arterial vasospasm without evidence of coronary artery stenosis on angiography [9-11].

## Case presentation

In 2007, a 58-year-old white male with no history of tobacco use, hypertension, diabetes, or hypercholesterolemia, and no family history of coronary artery disease, was found to have a large solid right renal mass and associated tumor thrombus of the inferior vena cava and right atrium. He underwent right radical nephrectomy and tumor thrombectomy performed under extracorporeal circulation. Cardiac function was investigated, including coronary angiography and transthoracic and transesophageal echocardiography. These studies documented normal cardiac function and normal morphology of the coronary arteries. Pathological examination of the surgical specimens demonstrated pT3cN0Mx conventional (clear cell) renal carcinoma, Fuhrman Grade 3 with negative surgical margins. A regular follow-up program was started. A few months later, hepatic and contralateral renal metastases were detected. The patient started treatment with the tyrosine kinase inhibitor (TKI) sorafenib on a dose-escalation protocol. Treatment led to an initial partial response followed by stable disease for 6 months. During December 2008, in a setting of stable disease, he underwent hepatic resection and enucleation of the left renal mass. The hepatic and renal masses were confirmed to be metastases. Early radiological assessment after surgery showed no residual hepatic or renal disease, but possible involvement of the mesenteric lymph nodes. Sorafenib treatment was therefore restarted at a standard dose of 800 mg/day. Treatment led to complete disease response within a few months, and was very well tolerated by the patient, who did not experience any of the typical adverse effects of the drug.

**Figure 1:**
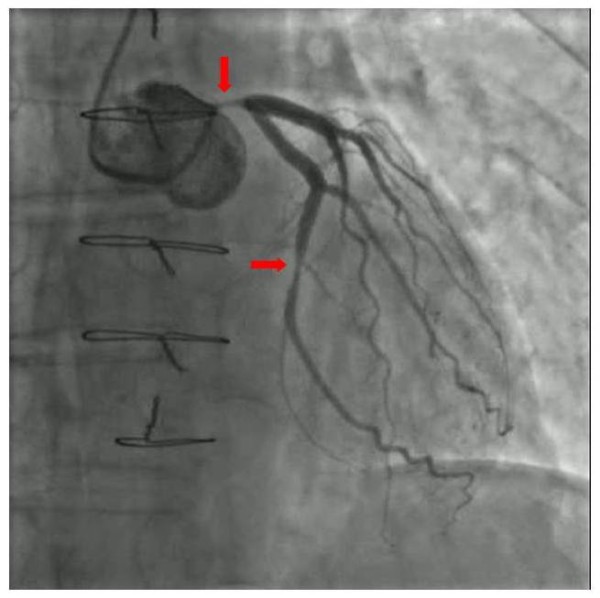
Coronary angiography showing critical sub-total occlusion of the common trunk of the left coronary artery and circumflex artery.

During February 2011, after 30 months of sorafenib treatment (6 months at 1600 mg/day and 24 months at 800 mg/day), the patient started to complain of worsening chest pain precipitated by normal physical exercise. A cardiac stress test showed exercise-induced ischemia. Coronary angiography showed critical sub-occlusion of the common trunk of the left coronary artery and the circumflex artery (Figure 1). The patient subsequently underwent triple coronary artery bypass surgery and is now recovering and in good clinical condition. Sorafenib treatment has been discontinued.

## Discussion

TKIs are currently approved for the treatment of mRCC [1,2]. Sorafenib is a multikinase inhibitor, with activity against transmembrane KIT, FLT-3, VEGFR-2, VEGFR-3, and PDGFR-B receptors, and intracellular CRAF and BRAF receptors. These kinases are involved in several angiogenesis systems and intracellular signaling pathways, and their disruption is aimed at inhibiting tumor growth [12]. The clinical efficacy of Sorafenib in the treatment of mRCC was demonstrated in a randomized, double-blind, placebo-controlled, phase III trial (TARGET) involving 903 patients who were resistant to previous therapy. An interim planned analysis of progression-free survival (PFS) showed a statistically significant benefit of sorafenib treatment over placebo (5.5 vs 2.8 months, p < 0.001). Consequently, a crossover was permitted from placebo to sorafenib. The difference in overall survival time was not statistically significant (19.3 vs 15.9 months), which can be explained by an important crossover effect. There was, however, a 28% reduction in the risk of death in patients receiving sorafenib [5,13]. Sorafenib may also be a suitable alternative treatment for selected naïve patients with clear cell mRCC [3]. A recent multicenter retrospective analysis of sequential treatment with sorafenib and sunitinib showed that initial treatment with either agent was associated with similar PFS times during first-line treatment (median PFS 8.4 months with sorafenib vs 7.8 months with sunitinib; p = 0.758). However, patients treated with sorafenib and then sunitinib appeared to have a slightly longer PFS times during second-line treatment than those treated with sunitinib and then sorafenib (median PFS with second TKI: 7.9 months vs 4.2 months; p < 0.001) [14].

Common toxicities due to sorafenib treatment include reversible skin rashes, hand-foot skin reaction, diarrhea, hemorrhage, hypertension, and laboratory findings such as leucopenia, hypophosphatemia, and elevated pancreatic enzyme levels [3,4]. Previous reports suggest that cardiotoxicity is a rare side-effect of sorafenib [4,5]. Some studies have commented on the cardiovascular toxicity of TKIs and monoclonal antibodies, in particular tumor angiogenesis inhibitors [6-8,15-18] (Table 1). The clinical events associated with cardiotoxicity include hypertension (8-45 % overall; 4-16 % grades 3 or 4) [3], LVEF dysfunction and/or CHF (<1 %-33.8 %) [5,7], and ATEs (3.3 % overall; 2 % high-grade with bevacizumab and 1.4 % high-grade with sunitinib and sorafenib) [6,8]. Several authors have concluded that LVEF dysfunction and/or CHF is more frequent in patients with a history of hypertension or coronary artery disease than those without, even though a careful cardiac assessment at baseline was not always reported.

**Table 1:** Data describing the main cardiotoxic events reported in clinical trials of tumor angiogenesis inhibitors

**Reference**	**Drug**	**N of pts**	**Events**	**Types of events**
**Ranpura V et al. [**[6]**] Meta-analysis of 20 RCTs**	Bevacizumab in pts with a variety of advanced solid tumors	1853	61 (3.3 %)	ATE (all-grade)
		5558	111 (2.0 %)	ATE (high-grade)
		2322	34 (1.5 %)	Cardiac ischemia
**Richards CJ et al. [**[7]**]**	Sunitinib	6936	186 (4.1 %)	Congestive heart failure
**Choueiri TK et al. [**[8]**]Meta-analysis of 10 studies**	Sunitinb and Sorafenib in pts with advanced cancer	9837	122 (1.4 %) 1.3 % for Sunitinib; 1.7 % for Sorafenib (NS)	ATE
**Chu TF et al. [**[15]**]**	Sunitinib in pts affected by GIST	75 36 75	8 (11 %) 6 (8 %) 10 (28 %) 7 (19 %) 35 (47 %)	Cardiovascular events Congestive heart failure LVEF reduction (at least 10 %) LVEF reduction (15 % or more) Hypertension
**Telli ML et al. [**[16]**]**	Sunitinib in pts affected by mRCC and GIST	48	7 (15 %)	Heart failure
**Di Lorenzo G et al. [**[17]**]**	Sunitinib in pts affected by mRCC	175	66 (37.7 %) 17 (9.7 %) 12 (6.9 %) 33 (18.9 %) of which 12 (6.9 %)	Hypertension G1-2 Hypertension G3 LVEF dysfunction Cardiac abnormalities LVEF G3 and/or Congestive heart failure
**Schmidinger M et al. [**[18]**]**	Sunitinib (Su) and Sorafenib (So) in pts with mRCC	74	25 [11 in Su; 14 in So] (33.8 %) of which: 12 (16.2 %) 13 (17.6 %) of which 7 (9.4 %)	Cardiac event Biochemical signs and ECG changes only Clinical symptoms (angina, dyspnea, dizziness) Life-threatening clinical symptoms

The molecular mechanisms of TKI cardiotoxicity have not been extensively investigated, but seem to be related to the inhibition of kinases that have a crucial role in normal cardiovascular development [19]. The role of BRAF in the heart is not well understood. RAF1 seems to inhibit two pro-apoptotic kinases with ERK-independent effects: apoptosis signal-regulating kinase 1 (ASK1) and mammalian sterile 20 kinase 2 (MST2), which are both involved in oxidant stress-induced injury. If sorafenib disrupts the RAF1–ASK1 and/or RAF1–MST2 interactions, cardiotoxicity might be an even greater concern than if only the ERK cascade is inhibited [20]. To examine the in vivo role of Raf-1 in the heart, Yamaguchy et al. generated cardiac muscle–specific Raf-1–knockout (Raf CKO) mice with Cre-loxP–mediated recombination [21]. The mice demonstrated left ventricular systolic dysfunction and heart dilatation, related to a significant increase in the number of apoptotic cardiomyocytes and fibrosis. The molecular mechanism of ATE has not been well investigated, but it seems to depend on multiple actions of VEGF on vascular walls, and maybe also on components of the coagulation cascade. VEGF stimulates endothelial cell proliferation and promotes endothelial cell survival, thereby helping to maintain vascular integrity. VEGF-signaling inhibition leads to a decrease in the regenerative capacity of endothelial cells, and causes cell wall defects that expose pro-coagulant phospholipids on the luminal plasma membrane or uncover the matrix, favoring thrombosis. VEGF-inhibitors may also inhibit nitric oxide and prostacyclin, increase endothelial cell apoptosis, and promote pro-coagulant changes and proliferation of vascular smooth muscle cells. Finally, VEGF-inhibitors may increase the risk of ATE by increasing the hematocrit and blood viscosity via overproduction of erythropoietin [3,22].

The absence of cardiovascular risk factors or any personal or family history of cardiac disease in our patient increases the suspicion of a correlation between his cardiovascular disease and sorafenib treatment. His cardiac function had been investigated and found to be normal before starting sorafenib, because his primary surgery was performed under extracorporeal circulation. He did not develop hypertension (average blood pressure before and during treatment: 125/80 mmHg) or other risk factors during sorafenib treatment (blood glucose level about 86 mg/dl, total cholesterol level about 141 mg/dl, HDL cholesterol level about 53 mg/dl, LDL cholesterol level about 75 mg/dl, and triglyceride level about 90 mg/dl). He therefore developed at least two critical sub-occlusive lesions during the 30 months after the start of treatment, which is a very short time period, considering the usual time it would take for such lesions to develop in a 58-year old man without cardiovascular risk factors. The risks of cardiac ischemia/infarction during treatment with angiogenesis inhibitors or TKIs are reported to be very low (1.5 % for bevacizumab, <1 % for sunitinib, 2.9 % for sorafenib) [6,18,23]*.* These events are usually associated with the presence of cardiovascular risk factors, but few detailed data on coronary angiography findings have been reported. Coronary angiography was performed and found to be normal in 7 of 74 patients with symptomatic cardiac events [18]. The frequency of treatment for acute cardiac ischemia was reported to be higher in patients receiving sorafenib (2.9 %) than patients receiving placebo (0.4 %), with an overall low rate of acute cardiac events during a median follow-up time of 16 months [5,13]. In the Advanced RCC Sorafenib (ARCCS) expanded access program, ATE events, including cardiac ischemia, were not reported [4]. Three cases of coronary artery disease related to sorafenib therapy have been described in the literature, which were all due to arterial vasospasm without evidence of coronary artery stenosis on angiography, and were all associated with cardiovascular risk factors [9-11]. Details of these three cases are presented in Table 2.

**Table 2:** Descriptions of the three reported cases which developed coronary artery disease during sorafenib treatment

**Reference**	**Case**	**Disease**	**Types of events**	**Cardiovascolar risck factors**	**Coronarography**
Naib T et al. [9]	57 years-old patient	HCC	multiple coronary vasospasm	history of diabetes, hyperlipidemia, former tobacco use	Normal
Arima Y et al. [10]	65 years-old patient	mRCC	coronary artery spasm	arterial hypertension	Normal
Porto I et al. [11]	63 years-old patient	HCC	variant angina for spontaneous coronary spasm	history of diabetes and arterial hypertension,	Normal

The case presented here indicates that attention should be paid to the potential occurrence of occlusive coronary artery disease during treatment with TKIs, and that patients should be carefully monitored for the development of symptoms of coronary ischemia/infarction. Even though cardiotoxicity is widely reported and recognized as an important though not frequent toxic effect of treatment with sunitinib and other tumor angiogenesis inhibitors, there is currently no consensus regarding the prevention and management of these side effects.

## Conclusions

In conclusion, we have presented a brief overview of the available data on cardiovascular events in patients treated with TKIs, and of the potential for the development of occlusive coronary artery disease. Understanding the usefulness of careful cardiovascular monitoring might be important to prevent fatal cardiovascular events and avoid discontinuation of treatment for the underlying cancer.

### Consent

The patient has given consent for the publication of this report.

## Competing interests

The authors declare that they have no competing interests.

## Authors’ contributions

Pantaleo MA and Mandrioli A analyzed the data and drafted the manuscript. Saponara M, Nannini M, and Lolli C drafted the manuscript. Erente G analyzed the data. Biasco G critically revised the manuscript. All authors read and approved the final manuscript.

## Pre-publication history

The pre-publication history for this paper can be accessed here:

http://www.biomedcentral.com/1471-2407/12/231/prepub
